# LIMS and chromatographic data acquisition in the manufacturing environment

**DOI:** 10.1155/S1463924696000181

**Published:** 1996

**Authors:** Mary D. Hinton, Phillip R. Hinton

**Affiliations:** Applied Computer Systems 3540 Country Ct. N. Mobile Alabama 36619 USA


Journal of Automatic Chemistry, Vol. 18, No. 5 (September-October 1996), pp. 169-174

### LIMS and chromatographic data acquisition in the manufacturing environment

**Mary D. Hinton and Phillip R. Hinton, Jr.**
*Applied Computer Systems, 3540 Country Ct. N., Mobile, Alabama 36619, USA*

*Chromatographic analyses often make up the bulk of the tests performed in manufacturing laboratories. To fully automate a laboratory, the LIMS must work closely with the chromatographic data acquisition system. There are probably as many solutions to interfacing a data acquisition system to a LIMS as there are LIMS vendors. One solution that works well in a manufacturing environment is described in this paper. The authors explain what functions are needed in the front-end (the data acquisition end) and the back-end (the LIMS), and how the two systems can work together to manage the chromatographic laboratory data.*

### Introduction

Chromatographic analyses often make up the bulk of the tests performed in manufacturing laboratories. To fully automate a laboratory, the LIMS must work closely with the chromatographic data acquisition system. Typical features often discussed when describing data acquisition systems are: the chromatogram for each injection can be viewed in real-time; peaks can be identified and calibrated according to retention time windows; the data for each injection is saved in a file; and data is collected from a number of instruments simultaneously, using a single chromatographic workstation. This paper does not discuss these features, but, instead, it focuses on those functions which are needed to facilitate a smooth coupling between the data acquisition system and the LIMS. The paper describes an example from a manufacturing environment.

Experience shows that there are certain functions that are better to implement at the front-end (the data acquisition end) rather than the back-end (the LIMS end). The functions needed in the data acquisition end are:

(1) Maintain a database of chromatographic data.
(2) Save the complete sample run.
(3) Log in sample information.
(4) Store process peak data.
(5) Allow all the injections in a current run to be viewed simultaneously.
(5) Allow 'on-the-fly' peak renaming or peak identification of unassigned peaks.
(6) Allow retrieving of previously stored runs.
(7) Store methods, sequences, and calibrations in the database.
(8) Export the sample run information.

The data acquisition systems on the market today do not have all these functions, therefore it was necessary to develop data acquisition software that could provide the functionality needed. Rather than reinventing the wheel,

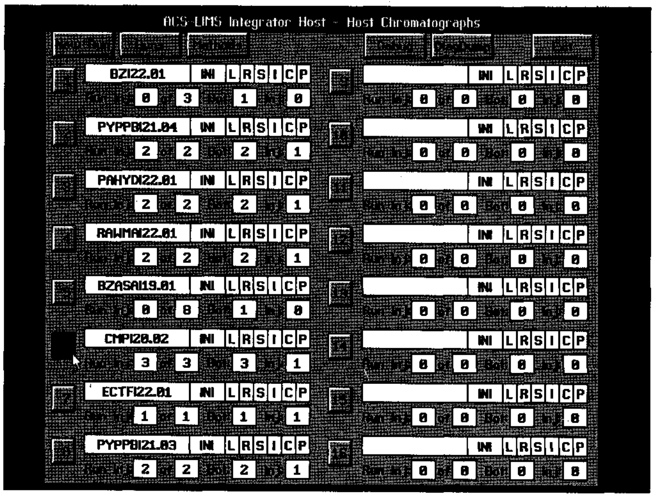

*Figure 1. ACS-LIMS integrator-host—main window.*

the approach taken was to rely on the chromatographic integrators already operating in the laboratory as the front-end to the new system. This was accomplished by connecting the RS-232 port of each integrator to a 16 port serial board interfaced to a PC, which acts as the host computer. Figure 1 shows the initial program screen displaying the status of each of the 16 integrators. The integrators do the base-line integration and display the chromatogram for each injection. This integrator-host software takes over the control of the integrators and maintains a database for manufacturing samples. The software obtains the peak counts in real-time from the integrators through the serial port and takes over the calibration and peak naming.

Commercially available chromatographic data acquisition systems do not maintain a database, but merely save each chromatogram into a separate file. The LIMS cannot automatically know which files go together to make up a complete sample run. Therefore, rather than saving individual injections into separate files, the data acquisition database should save complete run information, which includes all the injections that make up a run—the standards, samples, blanks, and references.

Along with the injections which make up a run, the sample being processed needs to be identified. Hence, before the sample is run, the sample should be logged into the data acquisition system and all the pertinent information for that sample entered. Figure 2 displays the log-in screen used in this data acquisition system.

The logged-in sample information includes the sample collection date and time, the sample material, the sample location point, any batch or lot ID, the method and

0142-0453/96 $12.00 © 1996 Taylor & Francis Ltd.

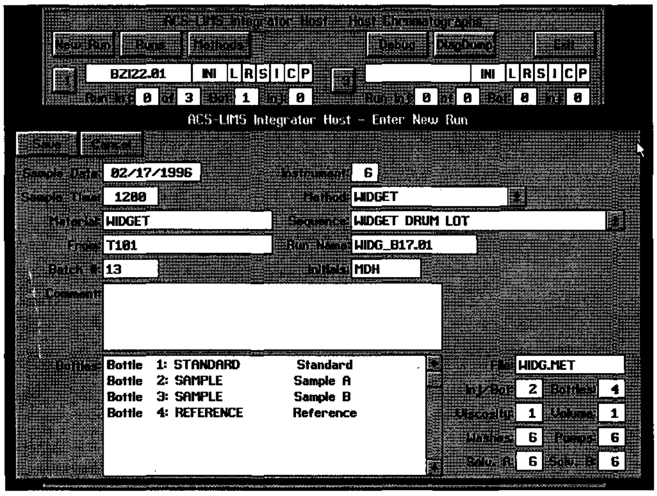

*Figure 2. New run control windows.*

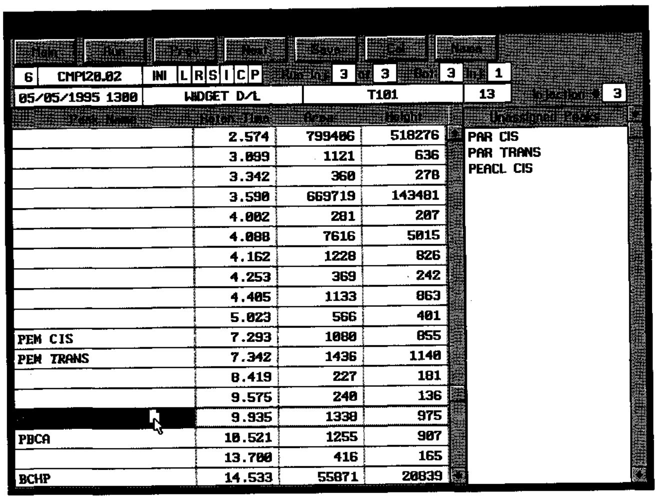

*Figure 4. Injection window.*

sequence to use, and the technician's user ID. The instrument number is automatically entered on this screen, with the instrument that the technician selected from the main screen. The method and sequence fields are both combo boxes, as represented by the arrow icon next to the field. When the technician enters the method field, a drop-down list of methods appears. The methods on the list were previously entered by the laboratory manager using the method editor section. The technician selects a method, then continues to the sequence field, which is also a combo box field. Only those sequences previously assigned for the selected method, using the sequence editor, are displayed in the drop-down list. After the method and sequence are selected, most of the remaining fields on the screen are automatically filled in, including the expected order and type of sample in each bottle. The types for the bottle can be: standard, reference, sample, or blank. The order and bottle type were previously assigned using the sequence bottle editor.

Once all the information is entered, the technician saves the sample information, and the program goes directly to the runs screen (see figure 3). The columns represent the individual injection results and the rows contain the assigned peak names. The technician starts the run by selecting the start button from the runs framer menu.

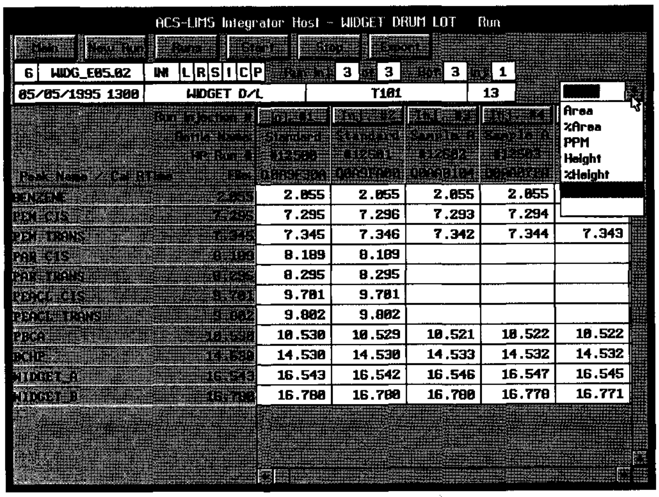

*Figure 3. Run information window.*

As the run progresses, the process peak information for each injection is saved. All the injections in a current run can be viewed simultaneously. When an injection is completed, the results appear on this screen. This program window makes it easy for the technician to compare the retention times for each standard peak with the retention times of the sample. Also, the current calibration is displayed to the right of the peak name. Previously stored runs can be retrieved at anytime.

The technician can view an individual injection by clicking on the injection number at the top of the injection column—figure 4 shows the injection window. The columns contain the counts for known and unknown peaks. The technician can name unassigned peaks or rename peaks 'on the fly' using the 'drag and drop' technique. If the technique determines that the chromatographic settings have drifted since the last calibration, he can recalibrate on a standard injection by selecting the CAL button from the framer menu.

The methods, sequences, and calibrations should also be stored. When the system is initially configured, all the methods files from the integrators are copied to the host computer in a separate directory for each integrator. Using the method list editor shown in figure 5, the laboratory manager enters the method name, the corresponding method integrator file, and any comments.

To edit a method, the user places the cursor in the method row. By selecting the PEAKS button on the framer menu, the PEAKS editor is displayed, as seen in figure 6. Here, the user creates or edits the components in a chromatogram, which include the known component peak names, the reference peaks, peak retention times, the retention time window, and any peak summing ranges required. The LIMS will maintain a calibration table for the named peak components for each chromatograph and for each method.

Similarly, selecting the SEQUENCE button from the method framer menu will display the sequence editor to enter sequence and bottle information.

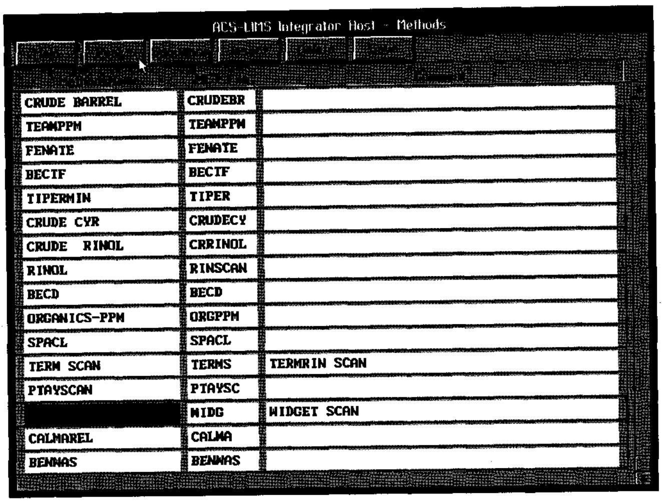

*Figure 5. Methods list window.*

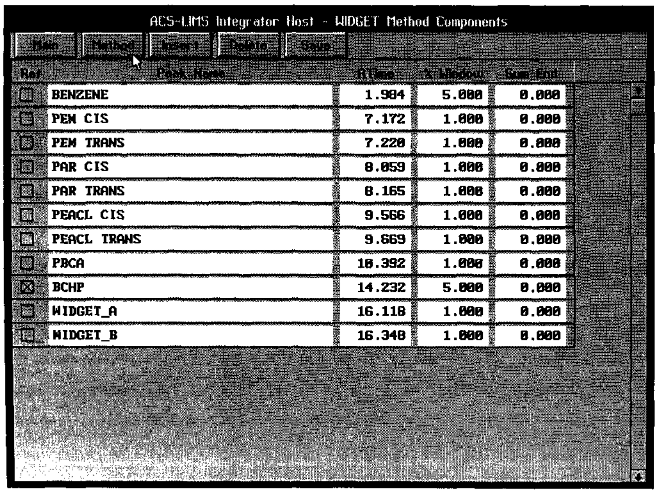

*Figure 6. Method components window.*

The next question to answer, was 'Where to physically store the database itself'. The goal was to allow the data acquisition system to continue operating locally even when the network is down, so it was decided to keep the data acquisition database on the host PC rather than on a network file server. This made it necessary to import the sample information into the LIMS as quickly as possible, so that process control and other departments can have access to the sample data without disturbing the technicians' workstation.

Now, the problem faced was how to transfer the data into the LIMS. There are numerous approaches that could be taken. One was to have the LIMS read directly from the host database, another was to have the host-integrator software write to the LIMS database on the file server at the same time as the host saves to the local database, and the third option considered was to have the host software write directly to files on the network file server. It was decided that since the host computer must keep up with data coming in from 16 integrators simultaneously, any extra CPU tasks on the host computer could adversely affect data acquisition. The simplest approach was taken, which is to have the host computer dump the run information to files on the network server to be picked up later by the LIMS.

This software was developed using another host-integrator program that already exported data in spreadsheet format. This part of the program could be modified to export the data to a LIMS. Now, as sample test data is collected, it is stored in the local database and then put in the host export queue, as sample test records. An export task runs in the background on the host PC at a low priority so that it does not interfere with data acquisition. This task is responsible for removing the sample test records from the export queue and putting the records into a LIMS import queue located on a network file server. If the network file server goes down, the host can continue to collect data and put sample test records into the host export queue while waiting for the network to come back online.

The sample information that should be imported into the LIMS follows:

(1) Each sample test record to be placed in the LIMS import queue will contain: a method name; a method sequence name; a sample material name; a sample source name (collection location); a sample collection date/time; the batch ID (or lot ID); the lab technician's initials (or ID); the number of injections per bottle; the number of bottles; and a bottle record for each bottle.
(2) Each bottle record will contain: a bottle type (which can be either sample, standard, reference, or blank); the number of injections for each bottle; and an injection record for each injection.
(3) Each injection record contains: the number of injection peaks; and a peak record for each injection peak.
(4) Each peak record contains: a peak retention time; a peak area; a peak height; and the identified peak name, if any.

Next, the LIMS end of the system needs to be considered, namely 'how to import the run information and what to do with it once it is imported'.

The LIMS needs to:

(1) Import the data from the data acquisition system into a usable LIMS format. The LIMS manager creates data entry forms to automatically import the peak information used in a test method. The forms should be flexible enough to set up any required calculations and any number of sample repetitions.
(2) Import standard values previously stored in the LIMS database for sample calculations.
(3) Maintain material specifications for chromatographic data.
(4) For final product, automatically generate the customer's certificate of analysis (COA) with the customer's specifications and all the test results required by the customer.

To import this data into the LIMS, one of the main concerns was to keep the LIMS program as easy to use as possible for the lab technicians. Basically, the technician should only have to push a few buttons on the LIMS screens to import the data. The LIMS manager who has a more in-depth knowledge of the system is responsible for setting up the test data entry screens for technicians' use.

The LIMS is considered first from the technicians' point of view and then from the LIMS manager's point of view.

### Technician's view of the LIMS

In figure 7, the technician selected test from the LIMS framer menu and a pull-down menu was displayed. From this menu, the technician selected import.

In figure 8, the import window displays all the sample files waiting to be imported into the LIMS. Each file is identified by the parameters stored in the sample test record used to uniquely identify the sample as it was logged into the data acquisition system.

To avoid having the technicians relog-in information in the LIMS that has already been logged into the data acquisition system, when a sample test record is selected from the import list, the LIMS database is searched. If the sample has been previously logged into the LIMS, the test window for this method is displayed right away. If it is not found, the LIMS log-in screen is displayed with all the sample information from the data acquisition system already entered into the correct fields. The technician then completes the log-in process.

Once the sample is logged in, the test method form is displayed, and the acquired data is then imported. Any calculations needed for this form are automatically performed. The test method form and calculations were previously set up by the LIMS manager.

Any peaks out of specification will be highlighted on the test method screen. The sample can be checked against any number of specifications. After reviewing the displayed data, the technicians save all the data on the form. This removes the sample test record from the LIMS import queue.

After all the tests for this sample are finished, the sample can be logged out. On the log-out screen, the status of each specification that the sample was tested against is given. The status tells whether the sample passed or failed each specification group. In our system, a sample may be multi-graded. For instance, if a sample represents final product, it can be checked against different customer specifications. One customer may only need a low-grade product, while another customer requires a premium-grade product. Multi-grading can help prevent high-grade product from being inadvertently sold as low-grade product.

The creation of COAs and other data reports is basically the final stage for the imported data. After the sample is logged out, and if the sample represents finished product, it becomes available for shipping. When the technician selects the ship button from the LIMS framer menu, the shipping window is displayed. This is shown in figure 9. Here, the technician enters bill-of-lading information. To eliminate errors, a pull-down menu or pick list of choices is displayed, wherever possible. For instance, in the product field, only those products purchased by this customer, will appear in the product pull-down menu.

After the bill of lading information is entered and saved, the technician selects the report button to automatically

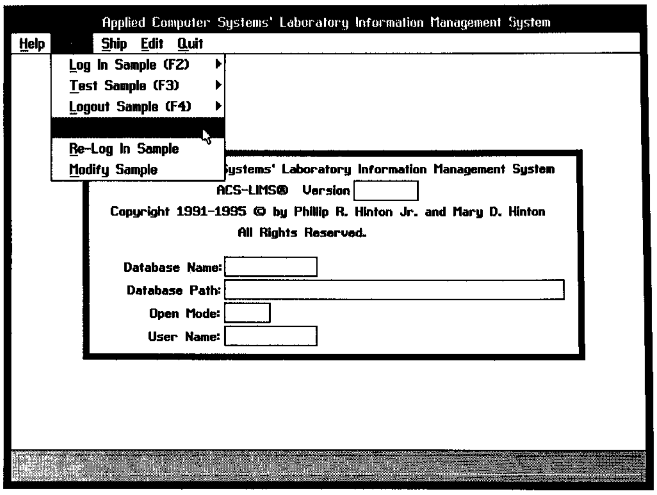

*Figure 7. ACS-LIMS pull-down many for the test section.*

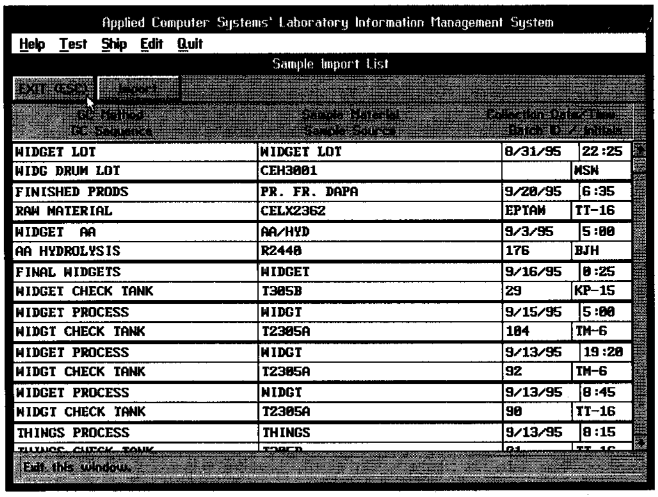

*Figure 8. List of imported sample data.*

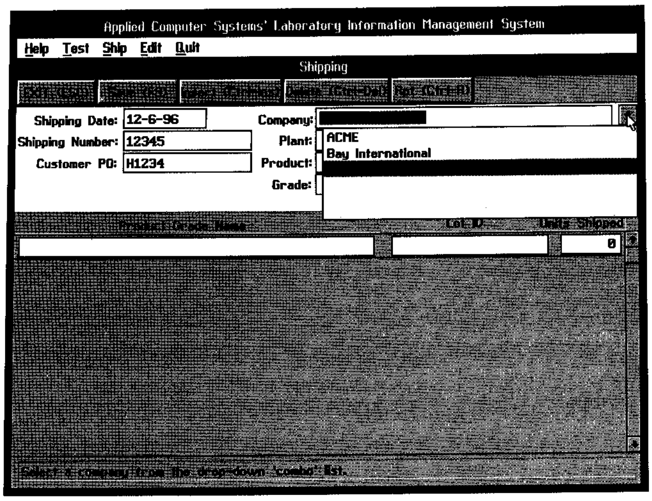

*Figure 9. Shipping window.*

generate a COA with the data results and the specifications for the individual customer.

### Manager's view of the LIMS

The LIMS manager function is very different from the laboratory technician. The LIMS manager manages the sample data using the edit section to:

(1) Create and maintain material specifications for chromatographic results.
(2) Design the test method data entry form for each test method.
(3) Import standards previously stored in the LIMS database into other test method forms that use the standard values for calculations.

After selecting edit from the framer menu, the LIMS manager will be prompted for a password, in order to continue. This section is used to enter all the laboratory information necessary to process samples. The edit pull-down menu displays various topics for editing as shown in figure 10. These topics include test methods, locations, material types, companies, containers, and user list. Each of these sections is further subdivided to enter more detailed information. For instance, using the material types topic, the manager enters all the materials tested in the laboratory, and the specifications for each material and test method. The specifications entered here are used to determine whether the material passes or fails. The LIMS maintains all revisions entered in the Specifications section.

The test method section, the first topic on the pull-down menu, allows the LIMS manager to create the data entry forms that will be used by the technicians. When test method is selected, a list of test methods appears, as seen in figure 11. If this is the initial configuration for the system, an empty list appears and the LIMS manager enters the names of all the test methods used in the laboratory. To create or edit a test method, the manager places the cursor in the row for the selected method, and selects edit from the test method tool bar. The manager can now create the test method form.

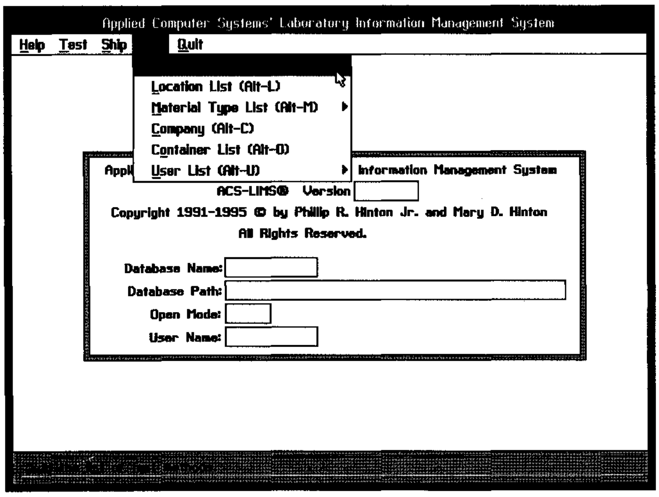

*Figure 10. Pull-down menu for the edit section.*

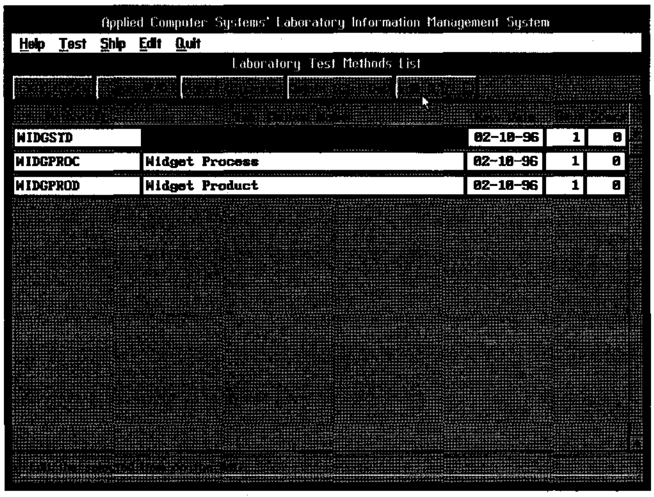

*Figure 11. List of test methods used in the laboratory.*

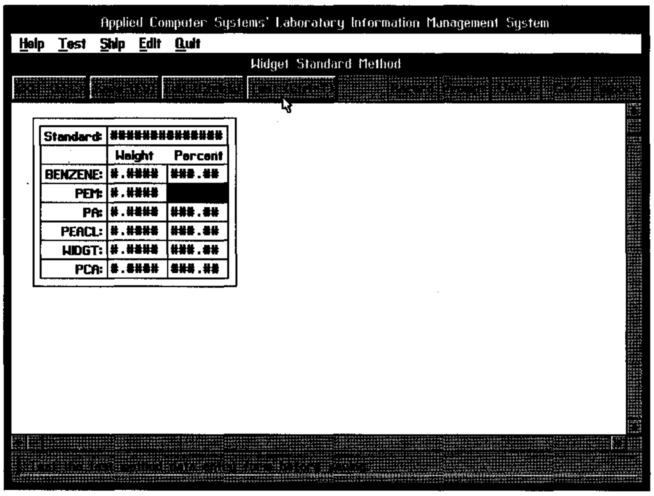

*Figure 12. Data entry form for 'widget' standard.*

Figure 12 is an example of a form created for entering the weights and concentrations of a standard composite. The values in this form can be imported into other test methods for calculating sample results.

Figure 13 displays a more complicated test form, one used to import data acquisition information. The import button on the test designers' tool bar is used to set up fields to import standard values—for example, the standard weights and percentage concentration from the previous form.

Using the edit button on the designer tool bar, the LIMS manager can assign a name and attach a formula or calculation to a field; an example is shown in figure 14.

The LIMS manager selects the test button from the designer's tool bar and enters test mode. As seen in figure 15, test data can now be entered. In this mode, any standard values will be inserted automatically, and calculations will also be performed automatically.

The form is also flexible enough to change the number of injections required. Using the insert button, the manager can add repetitions so he/she can see what the

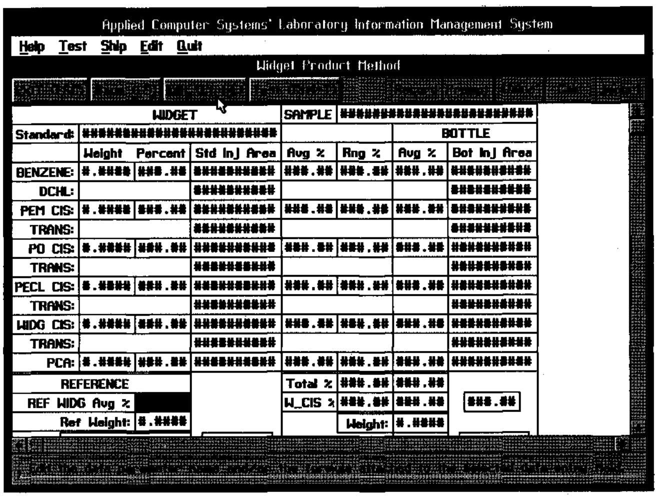

*Figure 13. Data entry form for importing acquired 'widget data'.*

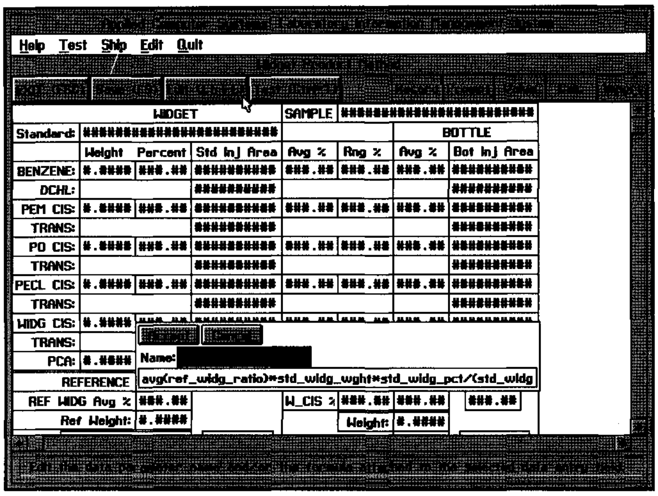

*Figure 14. An example of a data field with an attached formula.*

test screen will look like for more than one standard or sample. The number of repetitions can be saved on the form or the number of repetitions can be made part of the specification for the test method. Either way, the number of repetitions selected will appear on the test form for the technicians. The calculations will always be correct

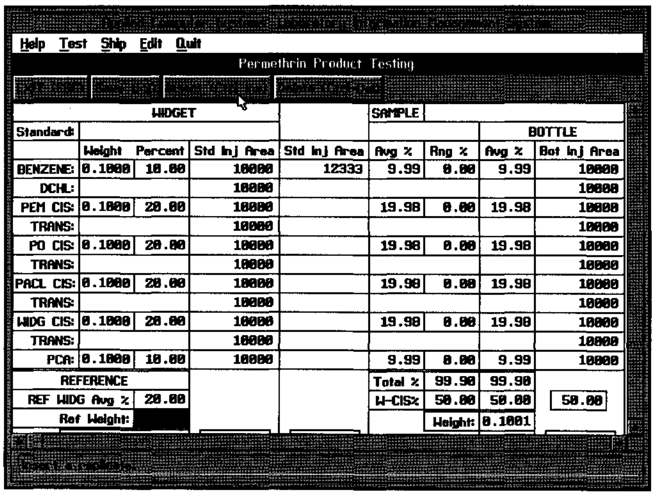

*Figure 15. Inserting a repetition in test mode.*

for the number of repetitions actually performed, but if the LIMS manager puts a specification on the number of runs, and the technician does not do the correct number of runs, the test will fail even though the result is within range. The LIMS manager has the choice whether or not to make the number of runs part of the specification. After the LIMS manager saves the form, it is made available to the technicians.

### Conclusion

This article describes a successful example of interfacing a chromatographic data acquisition system with a LIMS.

### References

1. Hinton, M., Hinton, P. and Williams, S., *American Laboratory* (January 1996), 51.
2. Hinton, M., *ACS-LIMS Integrator-Host Manual* (Applied Computer Systems, Mobile, Albama, 1995).
3. Hinton, M., *ACS-LIMS Manual* (Applied Computer Systems, Mobile, Alabama, 1995).
4. Hinton, M., *Laboratory Information Management Systems: Development and Implementation for a Quality Assurance Laboratory* (Marcel Dekker, New York, 1995), 156.

